# CdO/CdCO_3_ nanocomposite physical properties and cytotoxicity against selected breast cancer cell lines

**DOI:** 10.1038/s41598-020-78720-5

**Published:** 2021-01-08

**Authors:** R. P. Lefojane, B. T. Sone, N. Matinise, K. Saleh, P. Direko, P. Mfengwana, S. Mashele, M. Maaza, M. P. Sekhoacha

**Affiliations:** 1 Department of Health and Environmental Science, Central University of Technology, PO Box 339, Bloemfontein, 9300 Republic of South Africa; 2 Department of Pharmacology, University of the Free State, Bloemfontein, Republic of South Africa; 3 Department of Material Research, Nanoscience Laboratory, iThemba LABS, Cape Town, Republic of South Africa; 4 Flow Process and Rheology Centre, Faculty of Engineering and the Built Environment, Cape Peninsula University of Technology, Cape Town, Republic of South Africa

**Keywords:** Cancer, Diseases, Nanoscience and technology

## Abstract

Cadmium Oxide nanoparticles have the lowest toxicity when compared to nanoparticles of other semiconductors and they are not detrimental to human and mammalian cells, thereby making them candidates for targeting cancer cells. *Synadenium cupulare* plant extracts were used to synthesize CdO/CdCO_3_ nanocomposite using cadmium nitrate tetrahydrate 98% as a precursor salt. The resultant nanoparticles were characterized using scanning electron microscopy (SEM), transmission electron microscopy (TEM), X-ray diffraction (XRD), X-ray photoelectron spectroscopy, ultraviolet visible spectroscopy, and Fourier transform infrared spectroscopy (FTIR). The nanoparticles were then screened for effect on breast cancer cell lines (MCF-7 and MDA MB-231) and Vero cell line to determine their growth inhibition effect. Cytotoxicity effect was evaluated using 3-(4,5-Dimethylthiazol-2-yl)-2,5-diphenyltetrazolium bromide assay. XRD showed the peaks of monteponite CdO and otavite CdCO_3_ nanoparticles. TEM results showed irregular and spherical particles of varying sizes, whilst SEM revealed a non-uniform morphology. FTIR results showed peaks of functional groups which are present in some of the phytochemical compounds found in *S. cupulare,* and point to the presence of CdO. Annealed CdO/CdCO_3_ NPs showed selectivity for MCF7 and MDA MB231 in comparison to Vero cell line, thereby supporting the hypothesis that cadmium oxide nanoparticles inhibit growth of cancerous cells more than non-cancerous cells.

## Introduction

Cancer accounts for approximately 13% of all deaths annually worldwide^1^, and breast cancer is the most prevalent among women with 2 million new cases in 2018^2^. Breast cancer is characterized by the expression of oestrogen (ER), progesterone (PR), and HER-2 receptors^3^. These immunohistochemistry markers classify tumours as hormone receptor positive breast cancer. The tumours that do not express oestrogen, progesterone and HER-2 receptors are classified as triple negative breast cancer. The expression of markers determines the choice of cancer treatment. Hormone receptor positive breast cancer is treated with hormone interfering agents whilst triple negative breast cancer is treated with other forms of Chemotherapy. Treatment of triple negative breast cancer has the worst outcome when compared to other cancers due to the lack of molecular markers^4,5^. Generally, chemotherapy is associated with side effects such as killing of healthy cells and poor bioavailability of synthetic chemotherapeutic drugs, therefore research on chemotherapeutic agents that are more efficacious and less toxic to normal cells is vital^6^.

Nanoparticles are used as anti-cancer therapy in an effort to improve strategies for targeted delivery, in order to reduce toxicity to healthy cells. Metal nanoparticles have generated significant research interest in recent times due to their unique physical, chemical and biological properties, and have become the most effective research area^7^. CdO nanoparticles have the lowest toxicity when compared to nanoparticles of other semiconductors^37,38^. In addition, they have unique chemical, optical characteristics such as fluorescence, high resolution second harmonic generation and two photon-emissions, photoelectrochemical and electrical properties, which make them efficient anti-cancer agents^8^. In this regard, CdO/CdCO_3_ nanocomposite have been shown to induce apoptosis in cancer cells by inhibiting DNA repair and causing free radical-induced DNA damage, mitochondrial damage and disruption of intracellular calcium signalling, making them vital for targeting cancer cells. The major means by which CdO NPs act on cancer cells is via DNA, protein and cell wall damage^9^.

The synthesis of nanoparticles can be done using variant traditional methods which are costly, toxic and environmentally unfriedly^32^. The synthesis of nanoparticles is sub divided into the bottom up and top-down approaches^45^. In the bottom up technique, the synthesis of NPs is carried out by employing biological and chemical methodologies such as electrochemical methods, sono-decomposition and chemical reduction. Whilst the top down techniques comprise grinding, milling, sputtering, and thermal/laser ablation^34,45^. ‘The innovative method of green synthesis using plant extracts has been adapted in this study. The employment of green syntheses avoids the production of hazardous end-production by utilizing efficacious and environmentally friendly amalgamation techniques. This is achieved by using organic materials (plant extracts, fungi and algae) and various solvents. The processing and production of metal NPs in bulk using extracts from plants is easier in comparison to using other biological materials^34^.

*Synadenium cupulare* (Boiss. L.C) also known as *Euphorbia cupulare* or dead man’s tree, is a family of Euphorbiaceae. This plant is found in South Eastern Africa, Southern Mozambique, Swaziland, and South Africa. In South Africa, it is distributed in Kwazulu Natal, Mpumalanga and Limpopo Provinces. *S. cupulare* leaves and bark contain toxic latex, which has been used historically for treatment of toothache and infected wounds. Ground leaves are sniffed to treat headaches, flu and catarrh, or eaten for the treatment of asthma^10^. *S. cupulare* also possesses pharmacological activities; antibacterial, antioxidants, anti-inflammatory anthelmintic, anti-amoebic, antimicrobial and anti-cancer activities^6,11^. The administration of chemotherapy as cancer treatment kills healthy cells and its success rate in treating triple negative breast cancer is low. It has been hypothesized that CdO nanoparticles will selectively kill cancerous cells more than normal cells, hence the aim of the study was to determine effectiveness of CdO/CdCO_3_ nanocomposite against the selected cancer cell lines and its selectivity for breast cancer cells.

## Results

### Phytochemical analysis

*S. cupulare* leaf extract showed the presence of secondary metabolites such as tannins, flavonoids, saponins, and glycosides. The stem extract of *S. cupulare* showed the presence of phytosterols, pentose, tannins, glycosides, triterpenoids, saponins and flavonoids. Although anthraquinones and alkaloids were tested for in both the leaf and stem extracts, they showed a negative result.

### Characterization

**Figures 1:**
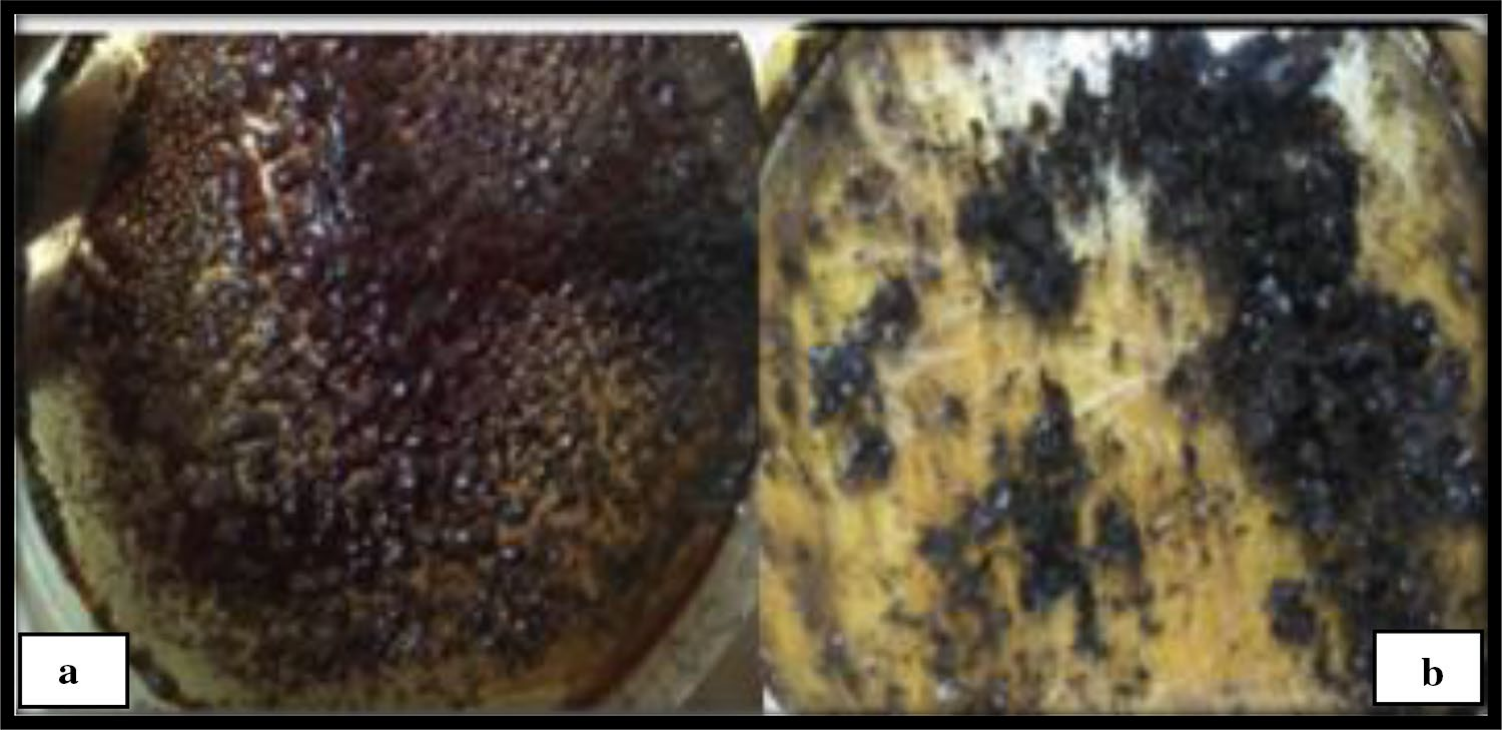
(**a**) Wet Cadmium oxide-extract moiety in the oven, (**b**) Dried CdO/CdCO_3_ NPs.

On heating, the cadmium-containing plant extract showed a reddish black color in the initial phase of drying (Fig. 1a), and progressed to a metallic shiny black, and crystalline as seen in (Fig. 1b).

**Figure 2:**
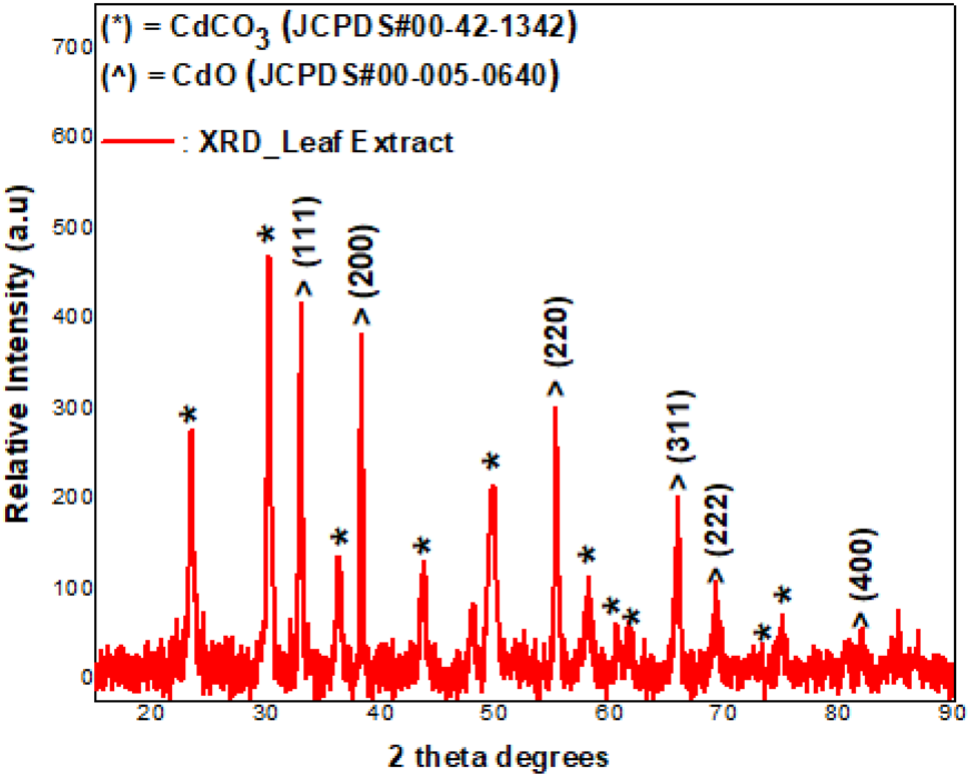
XRD of CdO/CdCO_3_ in the un-annealed state, synthesised using *S. cupulare* leaf extract.

XRD analysis of the Cd-containing leaf extract post annealing overnight at 200 °C showed the presence of Monteponite (CdO) and Otavite (CdCO_3_) (Fig. 2). CdO was found to have crystallized in the face-centred cubic phase (space group: Fm3m) while CdCO_3_ crystallized in the rhombohedral phase (space group: R-3c (167)), JCPDS cards number 00–042-1342. Reflections from the (111), (200), (220), (311), (222) and (400) planes were attributed to CdO as indexed in JCPDS #:00-005-0640. XRD diffraction of the sample dried at 70˚C show that the sample is crystalline with some degree of semi-amorphicity.

The crystallite size, *D*, of CdO and CdCO_3_ in the composite was determined using the Scherrer equation

1 $$D = 0.94\frac{\lambda }{\beta \cos \theta }$$

where 0.94 = Scherrer’s constant for spherical crystallites, *λ* = X-ray wavelength (1.5406 Å), β = FWHM (Full Width at Half Maximum) and θ = Bragg’s angle of diffraction. The average crystallite size for CdO was calculated as 36 nm while that for CdCO_3_ was 22 nm. (Table 1).

**Table 1:** XRD showing crystallite sizes derived using reflections from 5 (hkl) planes of CdO.

(hkl) CdO	Peak position 2θ (°) CdO	FWHM(°) CdO	D(nm) CdO	(hkl) CdCO3	Peak position 2θ (°) CdCO3	FWHM (°) CdCO3	D(nm) CdCO3
(111)	32.9989	0.2070	41.814	(012)	23.4411	0.3398	24.941
(200)	38.3063	0.2458	38.109	(110)	36.3245	0.3336	26.178
(220)	55.3034	0.2458	38.109	(110)	36.3245	0.3336	26.178
(311)	65.9624	0.3249	30.444	(202)	43.7324	0.4262	20.976
(222)	69.2658	0.3162	31.889	(116)	49.7852	0.6216	14.716
Av. Crystallite Size (nm)			36.35				21.93

**Figure 3:**
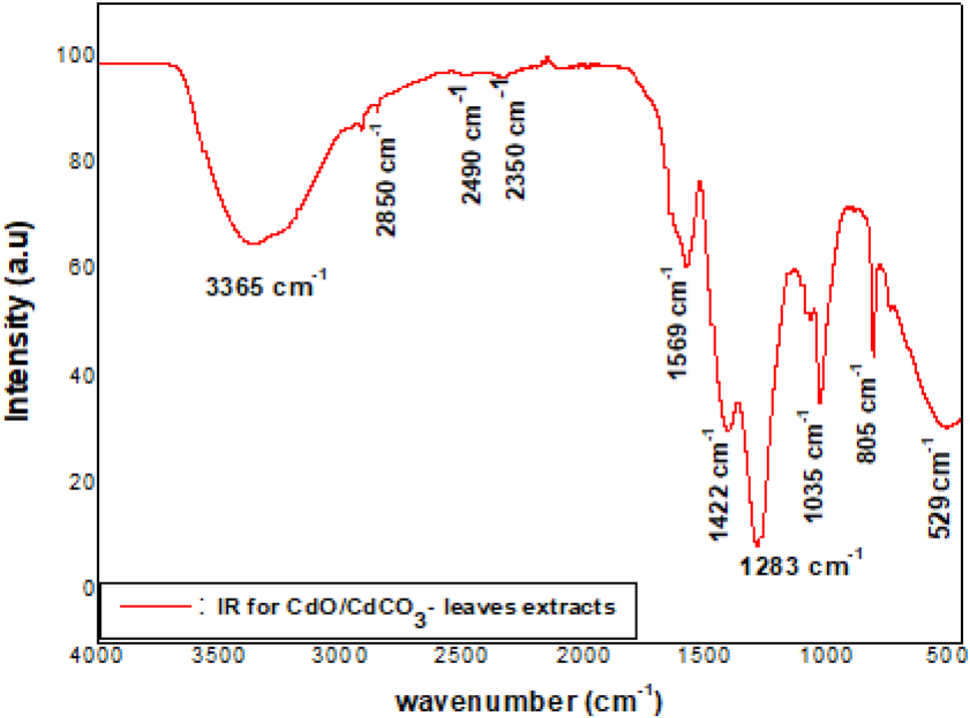
Infrared spectra of CdO/CdCO_3,_ in the un-annealed state.

Figure 3 shows the IR spectra of the CdO/CdCO_3_ containing extract, with peaks observed at 529, 805, 1035, 1283, 1422, 1569, 2350, 2490, 2850 and 3365 cm^−1^. The 1250–1310 cm^−1^ range is associated with C–O stretching aromatic ester, whilst the 1020–1250 cm^−1^ range depicts medium C–N stretching vibrations. The peak of medium intensity at 1569 cm^−1^ suggests the presence of N–O bond with stretching vibrations^24^. Peak 2350 cm^−1^ suggests the presence of an Isocyanate group (N=C=O stretching), whilst peak 2850 cm^−1^ which falls within 2800–3000 cm^−1^ range denotes medium C–H stretching vibrations^24^. The broad peak at 3365 cm^−1^ clearly points to the existence of surface-adsorbed water molecules in the dried sample. The fingerprint region for CdO lies in the 400–1000 cm^−1^ while that of CdCO_3_ is clearly evident with the 1422, 1569, 2490 cm^−1^ peaks especially^40^, all of which originate from complex deformations of the CdO and CdCO_3_ molecules^24^.

#### Morphological analysis

**Figure 4:**
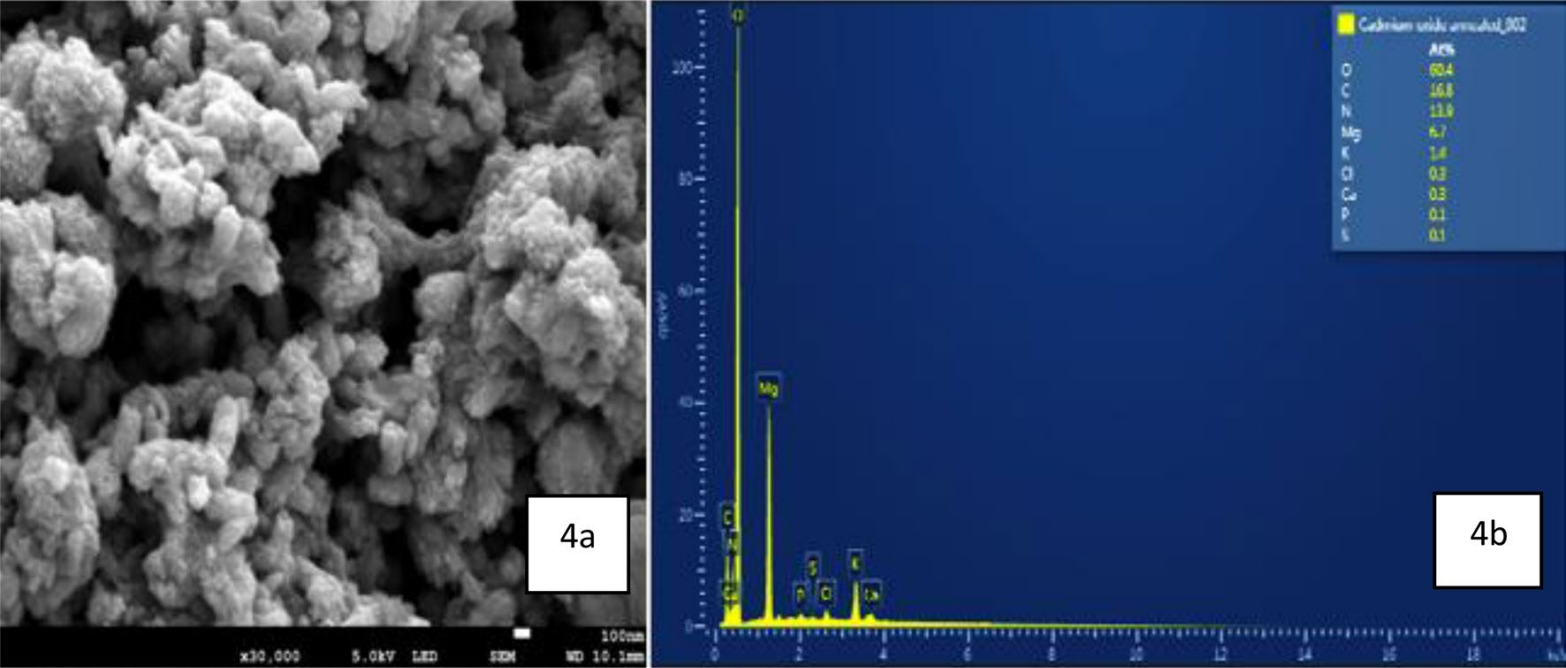
(**a**) SEM image of powder containing CdO/CdCO_3_ showing agglomeration and the presence of spherical structures, (**b**) SEM EDX spectrum showing different elements.

**Figure 5:**
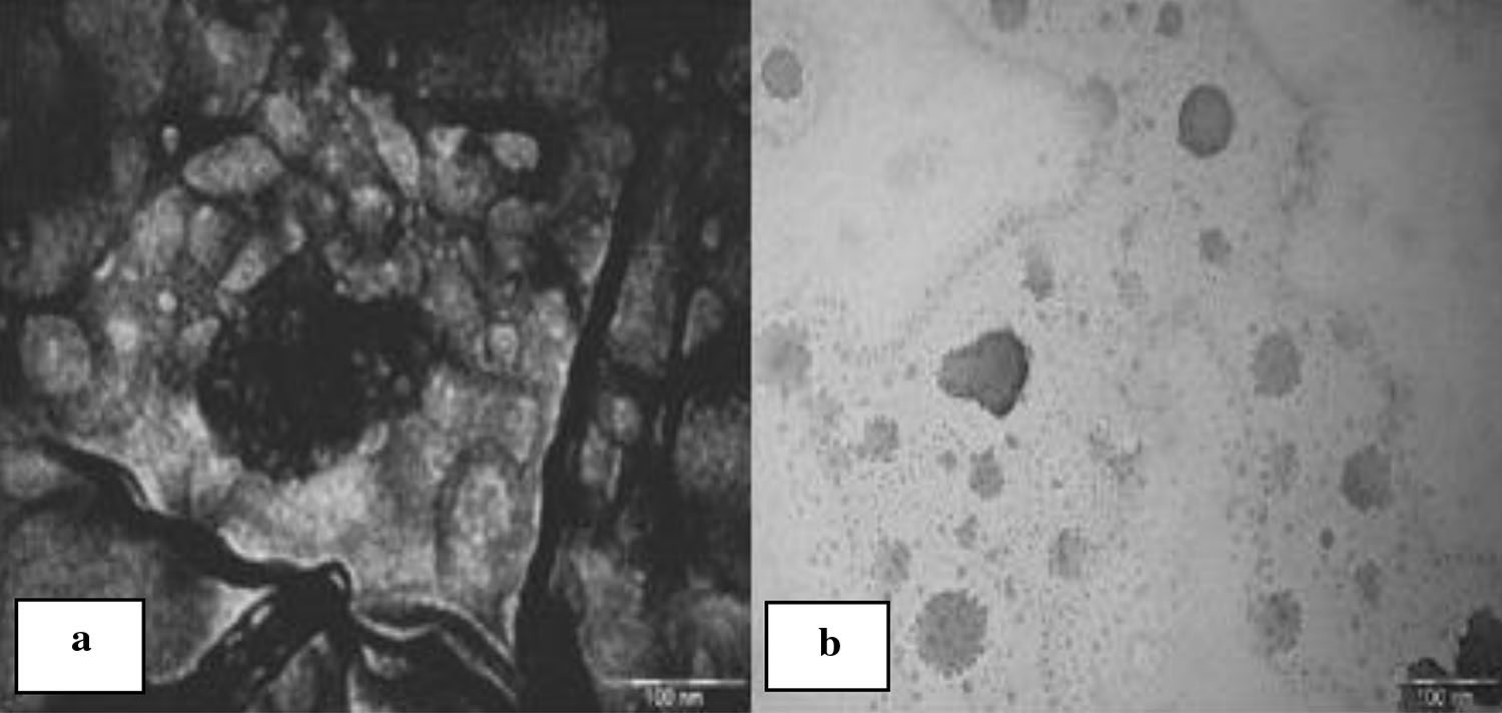
(**a**) Transmission Electron Microscopy image of annealed cadmium oxide nanoparticles showing spherical shapes, (**b**) unannealed nanoparticles showing non-uniform morphology.

The morphology of the synthesized CdO/CdCO_3_ NPs was characterised by SEM. CdO/CdCO_3_ nanoparticles showed agglomerated spheres with surface bound rod-like structures as seen in Fig. 4a. SEM EDX ( scanning electron microscopy with Energy Dispersive X-Ray ) spectrum showed the presence of oxygen, carbon, nitrogen, magnesium, potassium, chlorine, calcium, phosphorus, cadmium and sulphur in Fig. 4b. Figure 5a, b show morphology images obtained from TEM. Annealed nanoparticles showed irregular, spherical shapes of varying sizes at the scale of 200 nm, whilst unannealed nanoparticles showed an agglomeration of non-uniform shapes.

#### Chemical States determination via XPS

**Figure 6:**
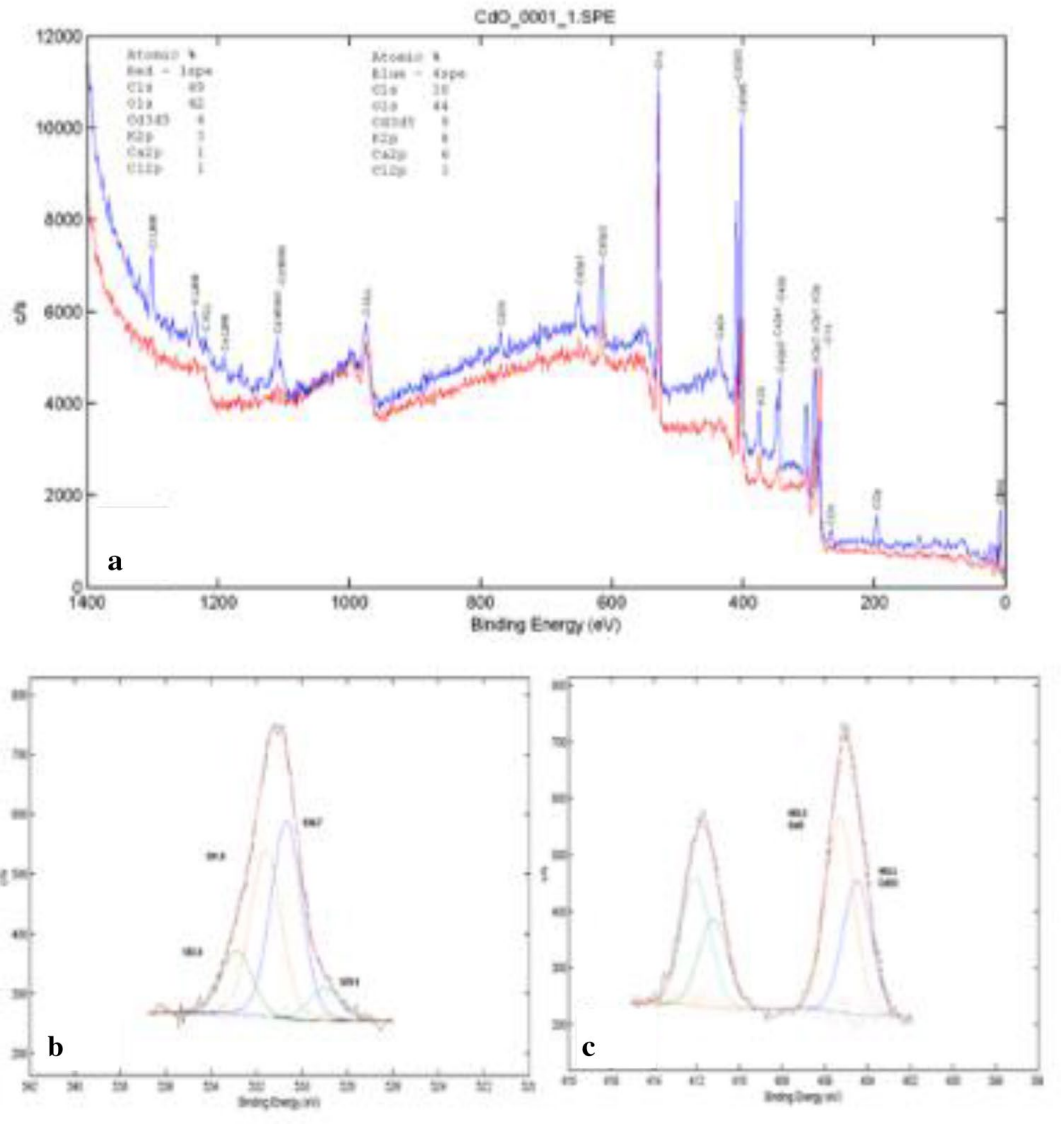
XPS (**a**) wide scan survey of the CdO-containing samples, (**b**) High resolution spectrum denoting the existence of O^2^ , and (**c**) High resolution spectrum denoting the presence of Cd^2+^ as in CdO.

Surface analysis to determine the oxidation states of annealed CdO/CdCO_3_ nanocomposite using XPS was carried out using a PHI 5000—Scanning ESCA XPS Microprobe. Wide scan survey and High Resolution spectra were done with a 100 µm, 25 W, 15 kV, Al monochromatic X-ray beam. The C 1 s peak (285 eV) was used as a reference for the binding energies. The presence of chlorine in appreciable amounts in the wide scan survey (Fig. 6a) may be due to the phytochemical compounds found in *S. cupulare;* possible contamination of the precursors used during sample preparation or to a lesser extent sample handling. The high resolution spectra confirm the presence of Cd in the + 2 oxidation state, consequently Oxygen in the -2 oxidation state. These results point to the presence of pure CdO in the annealed samples.

#### UV Absorption spectra analysis

**Figure 7:**
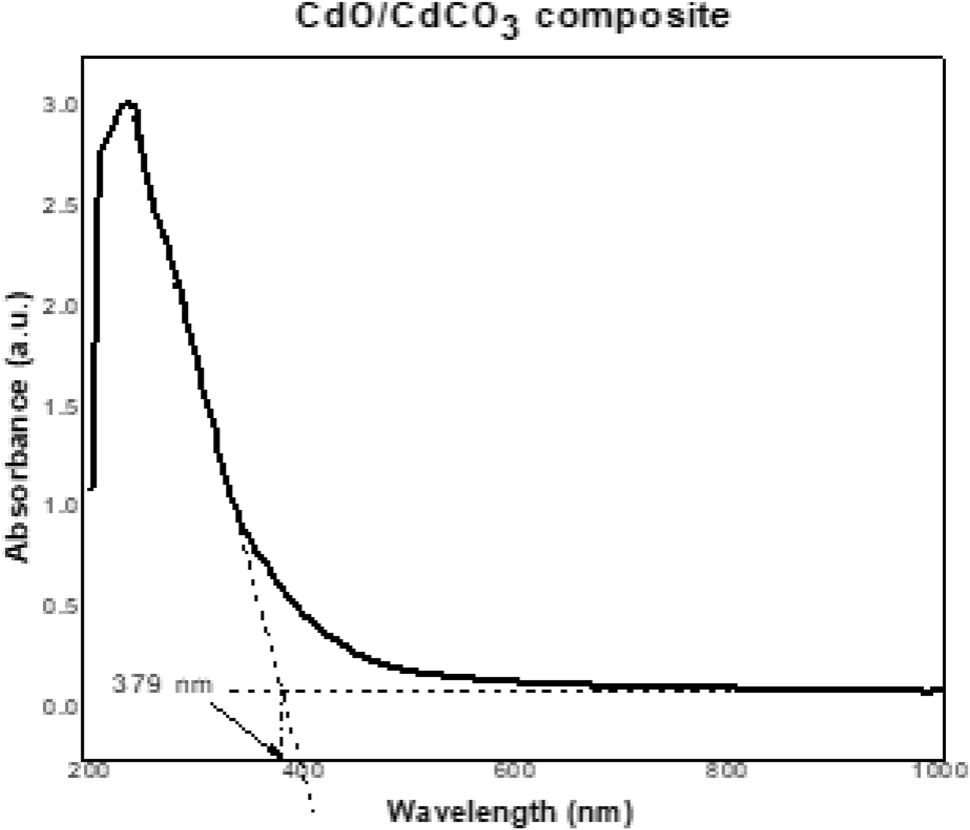
CdO/CdCO_3_ composite NPs UV–Vis spectra showing absorption edge at 379 nm.

Ultraviolet absorption spectrum of the CdO/CdCO_3_ composite is shown in Fig. 7. The absorption edge of the sample was observed at λ ~ 379 nm, suggesting a band gap of *E*_*g*_ (eV) = 1240 (eV.nm)/λ (nm) = 1240 eV.nm /379 nm = 3.27 eV. This band gap of 3.27 eV is reflective of the composite nature of the powder obtained as it lies between the band gap values obtained for pure CdO (*E*_*g*_ = 2.36 eV) and CdCO_3_ (*E*_*g*_ = 3.87 eV)^19,25,26^. The absorbance peak observed at 279 nm can be ascribed to water in which particles of the CdO/CdCO_3_ composite were dispersed^39^.

### Cell culture results

Cell growth inhibitory activity of *S. cupulare* leaf extracts; (hexane, dichloromethane, methanol, ethyl acetate, and water), as well as unannealed and annealed CdO/CdCO_3_ nanocomposites, was evaluated on MCF-7 (hormone receptor positive breast cancer cell line) and MDA-MB-231 (triple negative breast cancer cell line), breast cancer cell lines. Emetine and doxorubicin were used as standard drugs for cytotoxicity and anti-cancer activity, respectively. Selectivity was assessed on Vero cells, which is a kidney non-cancerous cell line.

#### Activity against the MCF-7 cell line

**Figure 8:**
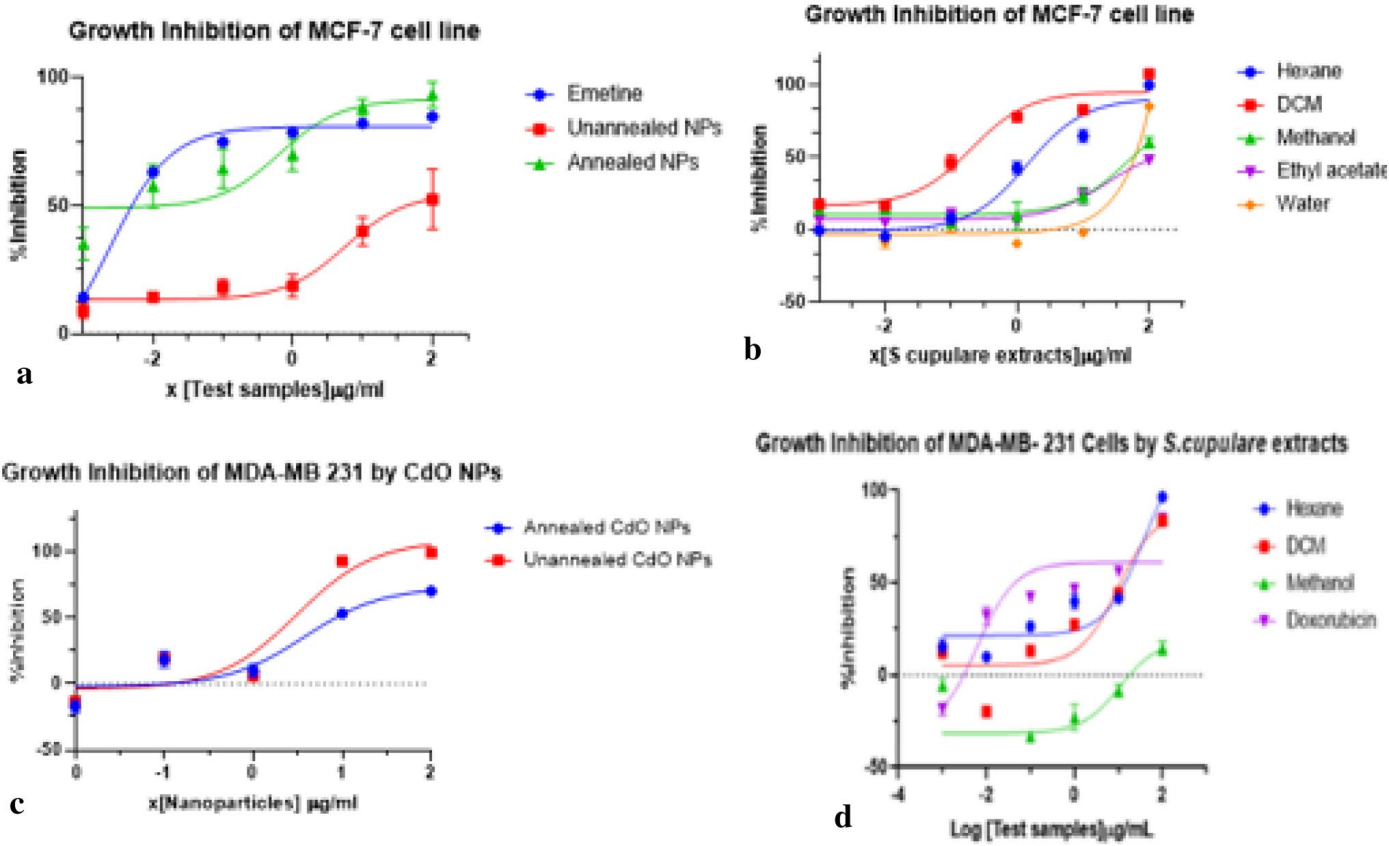
Growth inhibition of MCF-7 cell line by CdO/CdCO_3_ nanoparticles test samples (**a**) and *S. cupulare* extracts (**b**). Growth inhibition of MDA-MB-231 cells by *S. cupulare* -derived CdO/CdCO_3_ nanoparticles (**c**), and S.cupulare extracts (**d**).

The cytotoxicity standard drug, emetine, showed a high inhibitory activity with an IC_50_ value of 0.002 ± 0.532 μg/mL. The inhibitory effect of annealed and unannealed CdO/CdCO_3_ nanocomposite on MCF-7 cell lines is observed in Fig. 8a. Annealed CdO/CdCO_3_ nanocomposite showed a high inhibitory activity with an IC_50_ value of 0.652 ± 2.532 μg/mL whilst unannealed CdO/CdCO_3_ nanocomposite showed low inhibitory activity with an IC_50_ value that is above 100.

The hexane and DCM extracts of *S. cupulare* showed cell growth inhibitory activity with IC_50_ values of 1.427 ± 0.612 μg/mL and 0.202 ± 0.612 μg/mL respectively, while methanol and ethyl acetate extracts showed a decreased inhibitory effect on MCF-7 with IC_50_ values of 45.71 μg/mL and 58.71 μg/mL respectively. *S. cupulare* water extract showed cell growth inhibitory activity with an IC_50_ value above 100 μg/mL, which was classified as inactive. These results are shown in Fig. 8b.

#### MDA-MB-231 cell line

Doxorubicin showed **IC**_**50**_ value of 0.100 ± 0.532 μg/mL against the MDA-MB-231 cell line. Figure 8c shows growth inhibition of MDA-MB-231 cell line by *S. cupulare* extracts. The hexane and DCM extracts exerted cell growth inhibitory activity with **IC**_**50**_ values of 36.58 ± 3.54 μg/mL and 9.716 ± 3.06 μg/mL respectively. Methanol showed less inhibitory activity with IC_50_ value above 100 μg/mL. Figure 8d shows growth inhibition of CdO/CdCO_3_ nanocomposite, where annealed and unannealed CdO/CdCO_3_ nanocomposite showed **IC**_**50**_ values of 3.770 ± 0.530 μg/mL and 3.088 ± 0.637 μg/mL respectively.

#### Vero cell line

The inhibitory effect of emetine on Vero showed an IC_50_ value of 0.0074 ± 0.285 μg/mL, whilst annealed nanoparticles showed an IC_50_ value of 58.53 ± 0.285 μg/mL and unannealed nanoparticles showed IC_50_ value above 100 μg/mL.

#### Selectivity

Selectivity index (SI) depicts the differential activity of the test sample. The higher the SI value, the more selective the test sample is. An SI value less than 2 indicates general toxicity of the pure compound^27^. The SI is calculated as = IC_50_ of Vero/IC_50_ of Cell line (MCF-7 or MDA-MB-231)^28^.

Annealed CdO nanoparticles showed selectivity towards MCF-7 in comparison to Vero with an SI value of 90.04. IC_50_ value against MCF-7 was 0.65 μg/mL and 58.53 ± 0.285 μg/mL against Vero. SI of 90.04 shows high selectivity. Unannealed CdO/CdCO_3_ nanocomposite did not show selectivity towards either MCF-7 or Vero cell lines since both their IC_50_ values were above ≥ 100 μg/mL. Selective cytotoxicity was revealed on MDA-MB-231 by both annealed and unannealed CdO/CdCO_3_ nanocomposite in comparison to Vero cell line. Their SI values were both 15.5, which is indicative of considerable selectivity.

## Discussion

A powder containing CdO and CdCO_3_ nanoparticles was synthesized using the leaf water extract of *S. cupulare*. The qualitative analysis of *S. cupulare* leaf extract showed the presence of tannins, glycosides, saponins and flavonoids. Other metabolites such as phytosterols, pentose and triterpenoids were found in the stem extract. Metabolites found in plants play a huge role during green synthesis of metallic nanoparticles process. Hydroxyl and carboxyl groups found in secondary metabolites may act as reducing agents^16^. The reduced nanoparticles are capped and stabilized by functional groups such as alkaloids^17^. Therefore, the presence of alkaloids, saponins, and tannins in the leaf extract suggest a high concentration of reducing agents. The synthesised CdO/CdCO_3_ nanocomposite were reddish black/rusty in colour as seen in Fig. 1a, probably because of the natural colour of powdered CdO. The metallic shiny look of nanoparticles in Fig. 1b suggested the formation of CdO nanoparticles, which was confirmed by XRD. The presence of CdCO_3_ as observed could be a result of Cd^2+^ ions in solution interacting with carbonyls and –COOH groups within *S. cupulare* leaf extract. The FTIR spectra in Fig. 3 showed different peaks, which can denote a combination of bonds resulting from multiple bonds deforming simultaneously. The presence of the peak denoting the (O–H) can be due to the phytochemical composition of *S. cupulare*, which consists of tannins, glycosides and flavonoids. Their chemical structures all consist of an (O–H) group. Peaks observed in the High Resolution spectrum (Fig. 6c) at ~ 405.6 eV can be attributed to signals coming from Cd 3d_5/2_ core levels in CdO. The satellite peak of lower intensity observed at ~ 412 eV can be attributed to the Cd 3d_3/2_ core level and is a result of spin–orbit coupling. The spin–orbit doublet observed for both Cd core levels and the energy separation of ~ 6.4 eV between the satellite peaks in the binding energy region of 404–416 eV are typical of CdO at the nanoscale^18^. The peak observed at a binding energy of ~ 531.5 eV (Fig. 6b) can be ascribed to the O1s core level. The High-resolution spectra confirm the presence of Cd in the + 2 oxidation state, consequently O in the -2 oxidation states. These results suggest that CdO sample was pure in the region probed. The distinct CdO and CdCO_3_ peaks observed in XRD show clearly that pure CdO and CdCO_3_ aggregates exist alongside each other making it possible for us to observe Binding Energy peaks in XPS that specifically belong to CdO. Although the XPS spectrum carried out on CdO/CdCO_3_ material suggests the presence of pure CdO, it should be noted that the X-Ray rasters over a small area < 1 mm $\times$ 1 mm at a depth of 10 nm. It is possible therefore for the beam to fall precisely on a region of the sample which is purely CdO, hence we report on CdO/CdCO_3_, which is the actual composition of the sample.

The images of SEM and TEM CdO/CdO_3_ nanocomposite showed variant shapes as seen in Figs. 4a, 5a,b. The size, shape and composition of nanoparticles may vary as the result of condensation process that occurs during synthesis of nanoparticles, thereby leading to the formation of fine powders with irregular particles and shapes, hence the non-uniformity of the nanoparticles^20^. CdO/CdCO_3_ nanoparticles showed agglomerated spheres. Nanoparticle agglomeration is subject to numerous influences such as properties of the medium that the particles are suspended in. Agglomeration of nanoparticles occurs when nanoparticles collide and stick together by weak forces, which is a random procedure. Depending on the synthesis conditions and the surface chemistry, the nanoparticles tend to form soft or hard agglomerates^34,36^. The mechanism and parameters (pH, temperature, and salt concentration) employed in reduction also have the ability to control the size and stability of synthesised nanostructures.

SEM EDX spectrum revealed peaks of C, N, Al, Mg, P, S, Cl, K, Ca, Cd and O, with O showing the highest concentration. Adenosine Triphosphate (ATP), cell membranes and amino acids are the basic building blocks of every plant. Therefore Nitrogen (N), which is found in amino acids; phosphorus (P) which is found in ATP; and potassium (K) which is essential for a plant’s ability to metabolize, are always present in plants. Carbon (C) is present in the carbon dioxide that plants take from the air through diffusion whilst Oxygen (O) is found in water and its uptake into the plant is through osmosis. In addition, secondary nutrients such as magnesium (Mg), calcium (Ca) and sulphur (S) are needed for plant growth^29^, while aluminium (Al) is one of the four most common elements occurring in soils^30^. Rainwater, sea spray, dust, and air pollution are natural inputs of Chlorine (Cl) to soils, which is then transported as Cl^-^ across the plasma membrane^31^. *S. cupulare* which was used in the synthesis of CdO/CdCO_3_ has therefore contributed to the presence of these chemical elements in the synthesised nanoparticles.

CdO/CdCO_3_ nanocomposite showed stability. The stability was depicted by the sharp peak as observed in Fig. 7 of the ultraviolet–visible spectrum. UV–Vis absorption was obtained by dispersing the bulk of the sample in water and is representative of the bulk of the composite, whereas XPS probes just a small 5 × 5 micron area of the sample to a depth of 10 nm. XPS is therefore more likely to be less representative of the bulk than UV–Vis. The observation of pure CdO in XPS is thus merely localized and not representative of the bulk of the CdO/CdCO_3_ composite powders probed with UV–vis absorption.

Annealed CdO/CdCO_3_ nanocomposite showed a higher inhibitory activity on MCF-7 than unannealed CdO/CdCO_3_ nanocomposite, hexane and DCM extracts of the plant. In addition, annealed and unannealed CdO/CdCO_3_ nanocomposite showed selectivity for MDA-MB-231 cell lines in comparison to Vero. These results suggest that CdO/CdCO_3_ nanocomposite are not limited to one mode of action through receptor binding since MDA-MB-231 cell lines lack receptors. Literature shows that cadmium is a toxicant that has been classified as a probable human carcinogen. It is highly likely to damage the lysosome and cause DNA breakage in mammalian hepatocytes, and various cells and tissues^41,42^. Cadmium also disrupts mitochondrial function both in vivo and in vitro, and induces apoptosis^21^. Its oxide, CdO, has a low solubility in water (5 mg/L) but is soluble in vivo^40^. CdCO_3_ particles, on the other hand, are known to be insoluble in water as well as in vivo, thereby suggesting very limited ability to be toxic to human and mammalian cells^40^. Cytotoxic activity of the CdO/CdCO_3_ composite is therefore likely more attributable to CdO. The literature reports that the anti-cancer mode of CdO/CdCO_3_ nanocomposite is apoptosis^33,35^. When breast cancer cells are exposed to CdO/CdCO_3_ nanocomposite, programmed cell death increased^35,45^. However, other experiments show that the escalated apoptosis is related to the presence of p53 protein and mRNA in large amounts. In other instances, it is associated with chemically reactive oxygen species^43^. It is therefore acknowledged that apoptosis might not be the only mode of mechanism for cell death/cell inhibition by CdO/CdCO_3_ nanocomposites.

The national cancer guidelines state that an IC_50_ less than 30 µg/mL for a plant extract is considered active. With this as a benchmark, Hexane and DCM extracts showed a high inhibitory activity against MCF-7 and MDA-MB-231 breast cancer cells lines. It is deduced that the compounds responsible for activity are nonpolar since the activity was observed in nonpolar Hexane and DCM extracts. The inhibitory activity of hexane and DCM on the breast cancer cell lines support the historic use of *S. cupulare* as anti-cancer agent. Emetine exhibited inhibitory activity of IC_50_ value of 0.002 µg/mL, which is agreeable to ranges, reported in the literature^22^.

## Conclusion

CdO/CdCO_3_ nanocomposite were successfully synthesised using green Chemistry route. CdO/CdCO_3_ nanocomposite have a selective cytotoxic effect on breast cancer cells. In addition, the nanocomposite’s inhibitory activity on MDA-MB-231 shows that it is not limited to the mode of action of receptor binding, since triple negative breast cancer has no receptors. More cytotoxicity tests on normal breast cells should be performed to inform selectivity. The proportions of the concentrations of CdO/CdCO_3_ should also be calculated as this will add to literature of CdO and CdCO_3_ growth inhibition.

## Materials and methods

### Study area

The research study was conducted at the Central university of Technology. However, some experiments were conducted at the University of the Free State and iThemba LABS. The Study commenced in March and was completed in September 2019.

### Plant collection and extraction

Plants were collected from their natural habitat in Kruger National Park, Limpopo Province, South Africa. A Botanist at the Botany department, University of the Free State (UFS) authenticated the plant. The specimen was stored in the herbarium, UFS. The plant leaves and stems were washed with deionized water and dried at room temperature. Ground material (30 g) was mixed with 150 mL boiled de-ionized water (dH_2_0) and heated for 2–3 h at 60 °C whilst continuously stirring. The mixture was allowed to cool at room temperature and filtered using filter paper.

The plant leaves were sequentially extracted with hexane, dichloromethane, methanol and ethyl acetate. For each mixture, the ground plant material weighed 30 g and it was dissolved in 400 mL of the solvent. The mixtures were then extracted on the shaker for 48 h and the filtrates were concentrated using a rotary evaporator. The protocol was adapted from Mohsenipour et al.^12^ with some modifications.

### Phytochemical analysis

Phytochemical analysis was performed to determine the classes of compounds present in the plants. The methodology was adapted from Jeyaseelan and Jashothan^23^ with minor modifications.

#### Determination of phytosterols.

0.05 g of the powered plant material was weighed. To the powdered material, 10 ml of chloroform was added. 1 ml of concentrated H_2_SO_4_ was cautiously poured down the side of a test tube to a 0.5 ml of chloroform extract. An appearance of a reddish brown color in the chloroform layer was indicative of the presence of phytosterols.

#### Determination of pentose

40 mL of distilled water was added to 2 g of powdered plant material. The mixture was then filtered and 2 mL of hydrochloric acid containing phloroglucinol was added to 2 mL of the filtrate obtained. The mixture was then heated for 5 min. Formation of a red color was indicative of the presence of pentose.

#### Determination of tannins

20 mL of distilled water was added to 0.5 g of powdered plant material. The mixture was then boiled and filtered whilst still hot. The mixture was then treated with 3 drops of 0.1% of ferric chloride. A blue black precipitate was indicative of the presence of tannins.

#### Determination of glycosides

2 ml of acetic acid was added to 0.5 g of powdered plant material. The mixture was then treated with 1 drop of 0.1% of ferric chloride. 1 mL of concentrated sulphuric acid was added with caution to the mixture. The appearance of a brown ring was indicative of the presence of deoxy sugars.

#### Determination of triterpenoids

1 ml of chloroform was added to 2 mg of powdered plant material. 3 mL of concentrated sulphuric acid was then added with caution to the mixture. Formation of an interface with reddish brown colouration is indicative of the presence of triterpenoids.

#### Determination of anthroquines

12 mL of 10% HCl solution was added to 1 g of powdered plant material and boiled for 5 min. The mixture was then filtered and left to cool. 10 mL of chloroform was then added to the filtrate. The chloroform layer was then pour into a clean test tube and 10 mL of 10% ammonia solution was then added to it. The mixture was afterwards shaken and the formation of a rose pink colour at the top layer was indicative of anthroquines.

#### Determination of saponins

5 mL of distilled water was added to 0.5 g of powdered plant material, boiled and filtered. 3 mL of distilled water was then added to the filtrate. The mixture obtained was vigorously shaken for 5 min, after which frosting indicative of the presence of saponins was observed.

#### Determination of flavonoids

10 mL of ethyl acetate was added to 0.5 g of powdered plant material and heated for 3 min, allowed to cool then filtered.1 mL of dilute ammonia solution was then added to 5 mL of the filtrate. Upon vigorously shaking the resultant mixture a yellow precipitate, indicative of the presence of flavonoids, was observed.

#### Determination of alkaloids

2 mL of 1% HCl was added to 0.2 g of powdered plant material. 1 mL of Meyer’s reagent was then added to the mixture, followed by 1 mL of Drangendorff reagent. The appearance of an organic precipitate was indicative of the presence of alkaloids.

### Synthesis of CdO/CdCO_3_ nanocomposite

A volume of 100 mL of the *S. cupulare* leaf extract (pH = 5 and a temperature of 23 ˚C) was mixed with 5 g of cadmium nitrate tetrahydrate 98%. 5 g of salt was used to obtain a ratio of 1:20.The mixture was left to stir at 60–70 °C on a hot plate for 24 h. The mixture was transferred into a petri dish, and it was dried in the oven at a temperature of 70 °C until dry. The protocol was adapted from Yasir et al.^13^ with minor modifications.

### Annealing

The dry extract CdO-containing moiety was annealed in a furnace set at 200 °C for a period of 10 h.

### Characterization

X-ray diffraction (X-Ray diffraction model Bruker AXS D8 advance) with monochromated Cu Kα radiation of wavelength 1.5406 Å̊ operating at a current of 40 mA and a voltage of 40 kV in the Bragg–Brentano geometry, was used to characterize the crystallographic structures of the nanoparticles^14^. XPS PHI 5000 – Scanning ESCA XPS Microprobe was used for chemical analysis of CdO/CdCO_3_ nanocomposite. The infrared spectrum of the solid CdO/CdCO_3_ nanocomposite was recorded by Shimazdu FTIR spectrometer to identify organic and any inorganic material present. The particle shape of CdO/CdCO_3_ nanocomposite was determined by TEM and SEM. UV–VIS spectroscopy Shimazdu 1800 double beam UV–VIS was used for absorbance measurements.

### Screening of the nanoparticles for cytotoxicity

#### Cell culture

The culture environment was maintained at 37 °C in humidified, concentrated CO_2_ (5%) atmosphere. Cells (MCF-7, MDA-MB231, and Vero) were grown in DMEM media, supplemented with 10% serum (FBS), and incubated in the culture environment.

Trypsinization was performed when the cells reached approximately 90% confluency. Aliquots of 2 mL of warm (37 °C) trypsin–EDTA solution were added for 2 min to detach the cells. Then trypsin–EDTA was neutralized by adding equal amounts of complete medium^15^.

The cell viability was determined by using trypan blue staining solution, and cell concentration was counted by an automatic cell counter (Invitrogen). The cell suspension of 1 × 10^5^ cells/mL in aliquots of 100 µl was seeded in a 96-well plate, after which 100 µl growth medium was added to each well, followed by incubation at 37 °C in humidified 5% CO_2_ atmosphere for 24 h.

Following a 24-h incubation period, medium was aspirated, and the cells were treated with 100 µl of a range of dilutions (100–0,001 µg/mL) of nanoparticles and other control samples, in triplicates. Aliquots of 100 µl of media were added to make a final volume of 200 µl. The plates were incubated for 48 h. Cell growth and metabolic activity were measured using the MTT assay as described by Mossman et al. 1983. Excel and Prism Graph Pad 8 were used to analyze growth inhibition activity.
